# The Influence of Light on Reactive Oxygen Species and NF-кB in Disease Progression

**DOI:** 10.3390/antiox8120640

**Published:** 2019-12-12

**Authors:** Naresh Kumar Rajendran, Blassan P. George, Rahul Chandran, Ivan Mfouo Tynga, Nicolette Houreld, Heidi Abrahamse

**Affiliations:** Laser Research Centre, Faculty of Health Sciences, University of Johannesburg, P.O. Box 17011, Johannesburg 2028, South Africa; naresh.r84@outlook.com (N.K.R.); blassang@uj.ac.za (B.P.G.); rahulc@uj.ac.za (R.C.); ivant@uj.ac.za (I.M.T.); nhoureld@uj.ac.za (N.H.)

**Keywords:** photobiomodulation, reactive oxygen species (ROS), nuclear factor kappa-light-chain-enhancer of activated B cells (NF-кB), cancer, diabetes, wound healing

## Abstract

Reactive oxygen species (ROS) are important secondary metabolites that play major roles in signaling pathways, with their levels often used as analytical tools to investigate various cellular scenarios. They potentially damage genetic material and facilitate tumorigenesis by inhibiting certain tumor suppressors. In diabetic conditions, substantial levels of ROS stimulate oxidative stress through specialized precursors and enzymatic activity, while minimum levels are required for proper wound healing. Photobiomodulation (PBM) uses light to stimulate cellular mechanisms and facilitate the removal of oxidative stress. Photodynamic therapy (PDT) generates ROS to induce selective tumor destruction. The regulatory roles of PBM via crosstalk between ROS and nuclear factor kappa-light-chain-enhancer of activated B cells (NF-кB) are substantial for the appropriate management of various conditions.

## 1. Introduction 

Reactive oxygen species (ROS) are formed by the fractional reduction of molecular oxygen and include, but are not limited to, superoxide anions, hydrogen peroxide, and hydroxyl radicals, all obtained from sequential oxidation–reduction processes involving nicotinamide adenine dinucleotide phosphate (NADPH) oxidase, lipoxygenases, or cyclooxygenases [1]. Unusually high levels of ROS can allegedly be used for cancer diagnoses, varying according to tumor type, and are potent signaling molecules in cancer, leading to nuclear damage, genetic instability, and tumorigenesis [2,3,4]. However, at non-cytotoxic levels, ROS act as secondary messengers with signaling roles in many physiological systems to activate programmed cell death, gene expression, and other cell signaling cascades [5]. Increased ROS production was observed in diabetes and diabetic complications, leading to oxidative stress. As a result, a series of cell death mechanisms were observed within the cell, finally leading to tissue and organ damage. Elevated levels of blood glucose appear to be the prime source of free radicals, unbalancing the pool of antioxidants and ROS. Therefore, the down-regulation of ROS production and targeting factors resulting in their increased generation may have a significant role in controlling diabetic complications [6,7]. 

ROS plays a pivotal role in the initial stages of wound healing by killing invading bacteria and other microorganisms. However, under chronic conditions, increased production of free radicals was observed, thereby inhibiting the proliferation and migration of key cell types and leading to delayed wound healing [8,9]. The regulation of certain redox transcription factors is dependent on the level of ROS. Nuclear factor kappa-light-chain-enhancer of activated B cells (NF-кB) was the first discovered redox-regulated transcription factor. NF-кB is a protein complex with multiple functions in immune, inflammation, cell growth, and survival responses. ROS are able to both activate and suppress NF-кB signaling pathways [10,11]. Photobiomodulation (PBM) is a modern therapeutic approach which results in beneficial outcomes and the modulation of various signaling pathways in the presence of light at a specific wavelength. Photodynamic therapy (PDT) uses a specific wavelength light to activate the photosensitizer to induce cell death in conjunction with molecular oxygen. 

Even though PBM is well-known for its cell-stimulating properties both in vitro and in vivo, clinical studies have been very mixed, and some contradicted non-clinical studies [12,13,14,15]; as a result, some clinicians consider PBM a very controversial therapy [16,17,18]. It is important to realize that the underlying cellular mechanisms of PBM are not fully understood [19,20]. Additionally, PBM treatment parameters vary, such as the wavelength, fluence, power density, pulse structure, and irradiation time. These are factors that preclude efficient clinical transition of PBM [21,22,23,24]. However, some studies reported the role of cytochrome c oxidase as an important chromophore in the cellular response to PBM [25]. A similar problem exists with PDT, in that it is not clinically accepted by many clinicians. Although photodynamic therapy (PDT) has a long history, there is a minimal amount of proven clinical research, making it difficult for it to be recognized as a first-line treatment approach in modern medicine. This review focuses on the effect of ROS on NF-кB activity, how ROS are affected by PBM/PDT, and its role in diabetes, wound healing, and cancer, respectively. 

## 2. Sources and Stimuli of ROS

ROS are oxygen intermediates with unpaired electrons; both superoxide and hydroxyl radicals are highly unstable oxygen radicals [26]. Experimentally, hydrogen peroxide is a simple peroxide radical involved in various signaling functions and is frequently used as a source of all oxygen-related free radicals [27]. Elevated levels of hydrogen peroxide effectively oxidize cysteine residues (Cys-SH) to cysteine sulfenic acid (Cys-SOH) or cysteine disulphide (Cys-S-S-Cys) in various proteins, such as kinases, phosphatases, and transcription factors. A well-established mechanism by which ROS regulate cellular functions is through the redox balance of cysteine residues [28]. 

**Figure 1:**
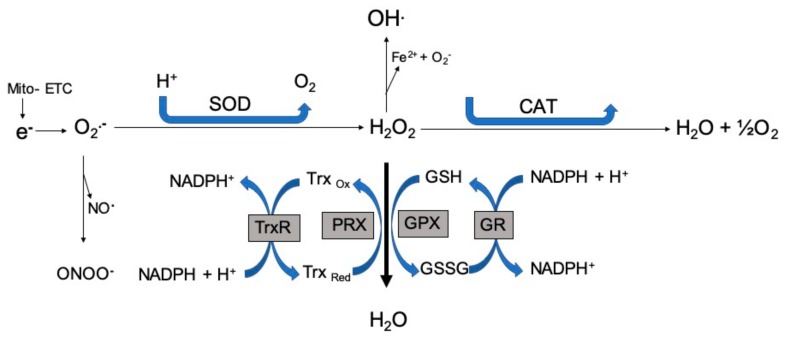
Free radical generation. Free radicals formed by the electron transport chain are immediately removed and converted to water through a series of enzymatic reactions. ETC: electron transport chain; SOD: superoxide dismutase; CAT: catalase; GPX: glutathione peroxidase; GR: glutathione reductase; PRX: peroxiredoxins; GSH: glutathione; GSSG: glutathione disulfide; Trx: thioredoxin; O_2_•^−^: superoxide; NO•: nitric oxide; ONOO^−^: peroxynitrite; H_2_O_2_^−^: hydrogen peroxide.

Mitochondria and NADPH promote endogenous ROS formation in cancer and reports have shown crosstalk between these two producers [29]. The mitochondrial oxidative generation of adenosine triphosphate (ATP) is a major source of free radicals. During the Krebs’s cycle, unpaired electrons are transferred to the electron transport chain (ETC), resulting in the production of superoxide anions [30]. About ten mitochondrial sites generate numerous superoxide anions through different mechanisms [31]. The reactions of the five complexes of the ETC (complex I to V) are involved in the production of ATP and free radicals as byproducts. The radicals produced from complexes I and III have various cellular signaling roles [32]. Both superoxide anions and hydrogen peroxides are constantly produced by complex III, with 80% released into the intermembrane space and the rest into the mitochondrial matrix. These ROS intermediate radicals are necessary for cell differentiation, proliferation, survival, and adaptive immunity responses [33]. In complex IV and during oxidative phosphorylation reactions, the movement of electrons results in the reduction of oxygen to water. Almost all of the generated superoxide anions are effectively quenched by manganese superoxide dismutase (MnSOD) to form hydrogen peroxide, which serves as an important precursor for other free radicals and acts as a secondary messenger with the ability to diffuse across the mitochondrial membrane, mediated by a specialized protein from the aquaporin family [33,34]. Other than the positive functions, mitochondrial ROS also have some deleterious ones, including the formation and progression of various cancer types like chronic lymphocytic leukemia, acute myelogenous leukemia, and breast cancer [35]. Figure 1 depicts the generation of free radicals and their fate in the ETC. Free radicals formed by the ETC are immediately removed and converted to water by a series of enzymatic reactions. 

### 2.1. Oxidative Stress and Cancer

Oxidative damage is linked to all phases of cancer, during which ROS act throughout its progression. Radicals generated in the mitochondria damage genetic material and produce mutations that initiate tumor progression due to the imbalance between ROS generation and antioxidant defence mechanisms [36]. Redox homeostasis is important to sustain normal and survival functions. Increased aerobic glycolysis and high levels of free radicals in cancer cells are linked to modifications in cell signaling pathways, which can be counteracted by antioxidant defence mechanisms [37]. ROS facilitate carcinogenesis by reversibly inhibiting certain tumor suppressors, such as phosphatase and tensin homolog (PTEN) and protein tyrosine phosphatases (PTPs), which enhance antioxidant expression and thus decrease ROS levels. However, high levels of ROS are beneficial during the developmental stages of tumors, promoting cancer vulnerability and death [38]. 

### 2.2. Oxidative Stress and Diabetes

Glucose metabolism is the mechanism behind the generation of oxidative stress. In a transition metal-dependent reaction, the enediol form of glucose is oxidized to enediol anion radicals, then converted to more reactive superoxide anions and ketoaldehydes. Through a dismutation reaction, superoxide anions are converted into hydrogen peroxides, which are precursors of highly reactive hydroxyl radicals. Under diabetic conditions, if these hydrogen peroxides are not degraded by glutathione peroxidase (GPx) or catalase (CAT), then the production of reactive hydroxyl radicals occurs in the presence of transition metals [39]. Superoxide radical formation during hyperglycaemia may also promote additional ROS formation through the oxidation of low-density lipoproteins [40].

Amadori products and advanced glycation end-products (AGEs) are emerging as major precursors of free radicals in diabetes. AGEs may fluoresce, produce ROS, and bind to specific cell surface receptors [41]. These generated AGEs bind to their receptors via the receptor for AGEs (RAGE) and deactivate enzymes, followed by the discharge of free radicals [42]. These enzymatic alterations may also quench and inhibit the anti-proliferative effect of nitric oxide (NO), which acts as a vital vasodilator in diabetic patients [43]. Altogether, the increased free radical production stimulates intracellular oxidative stress by AGEs, which upregulate NF-кB-controlled target genes [44]. In endothelial cells, ROS stimulates the hexosamine pathway and induces vascular complications in patients with high blood glucose levels [45]. The overexpression of the enzyme xanthine oxidase may contribute to the pathological condition, leading to type 2 diabetes. One of the mechanisms underlying this is the ability of xanthine oxidase to produce free radicals and oxidative stress [46]. 

### 2.3. Oxidative Stress and Wound Healing

The main processes involved in regulated wound healing are migration, adhesion, proliferation, neovascularization, remodeling, and apoptosis. Imbalance between antioxidants and free radicals with elevated ROS formation is often observed, followed by the induction of apoptosis. In chronic wounds, elevated free radicals coupled with reduced antioxidants results in the stimulation of pro-apoptotic transcription factors, caspase-3, and oxidative stress, leading to cell death and further delays in the healing process. The development of chronic impaired wound healing involves certain cell signaling reactions [47]. Fibroblast/keratinocyte cells with mutated mitochondrial DNA result in increased ROS generation, which affects nuclear transcriptional events by stimulating various signal transduction pathways and inducing cell cycle arrest, death, and oxidative stress processes [48,49]. In fibroblasts, mitochondria-generated ROS damages mitochondrial DNA and induces a persistent oxidative stress condition. Keratinocyte differentiation depends on the ETC for the accumulation of superoxide anions [50]. In contrast, in normal wound healing, minimal ROS levels mediate intracellular signaling for cell proliferation and collagen deposition. Low ROS levels and high antioxidant levels are essential for normal tissue repair [51]. 

### 2.4. Influence of Light on Oxidative Stress

Under two different conditions, light is used to either attenuate oxidative stress (as in the case of PBM) or generate excessive amounts of ROS (as in the case of PDT). PBM involves the use of non-ionizing radiation or light specifically in the visible and near-infrared regions of the electromagnetic spectrum for therapeutic applications and stimulation of various cellular mechanisms. When there is an imbalance between antioxidants and free radicals, PBM facilitates the displacement of free radicals and the reduction of the oxidative stress load on the organism [52]. The successful action of PBM depends on the activation of cytochrome c oxidase, which catalyzes the reduction of oxygen to water by increasing the mitochondrial membrane potential (MMP), ATP, cyclic adenosine monophosphate (cAMP), and NO [53,54]. NO is an essential signaling agent for the activation of certain cellular pathways. PBM increases the generation of NO by upregulating cytochrome c levels and/or by cleaving the metal complexes in cytochrome c [55]. The basic concepts and principles behind PBM are clearly described in the literature. It is understood that PBM acts on impaired or dysfunctional tissue through light and leads to mitochondria-mediated cellular processes, thereby inciting various mechanisms that result in a wide range of therapeutic functions [56]. Some studies proposed that light at wavelengths between 400 and 500 nm may generate free radicals through photosensitization, while ROS production by mitochondria was directly evident at longer wavelengths. PBM activates several transcription factors in the cytosol such as NF-kB, which trigger the transcription of genes and protect cells from oxidative stress [57]. 

**Figure 2:**
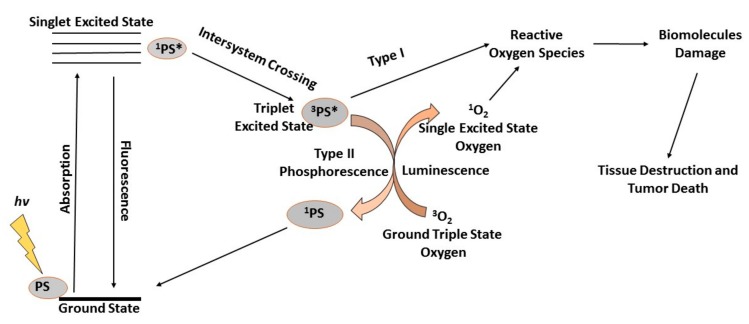
During photodynamic therapy (PDT), generated reactive oxygen species (ROS) kill the targeted tumor cells. PDT depends on the successful accumulation of photosensitizers (PSs) in tumor cells to initiate the process of irradiation of targeted areas for activation of the PSs. When irradiated with light of a specific wavelength, a photochemical reaction leads to the production of ROS in the presence of oxygen. When the PS absorbs the photon energy it is excited and jumps from the ground state to the excited singlet state and the energy is emitted back as fluorescence or heat via internal conversion. Should intersystem-crossing occur, the PS is converted to an excited triplet state, which can transfer electrons with cellular biomolecules, ultimately leading to the generation of ROS in a Type I reaction. Alternatively, the excited triplet state PS transfers its electrons to ground triplet state molecular oxygen (^3^O_2_), leading to the generation of an excited singlet oxygen (^1^O_2_) in a Type II reaction.

PDT is a minimally invasive treatment that is becoming commonly accepted as a potential therapeutic option for localized cancers [58]. PDT depends on the successful accumulation of photosensitizers (PSs) in tumor cells to initiate the process of irradiation of targeted areas for activation of the PSs (Figure 2) [59,60]. The wavelength of light should match the absorption properties of the PSs for effective ROS generation, either through direct electron abstraction (type I) or transfer from a substrate to oxygen in a highly reactive state (type II), killing targeted tumors and leaving the neighboring healthy cells unaffected [61]. Due to light inaccessibility to all areas of the body, PDT was previously limited to superficial conditions, leaving deeper diseased organs less affected. Due to the advancement in science and the medical use of fiber optics, PDT now has the potential to treat both superficial lesions and deeper-seated tumors, which were previously inaccessible. The recent development of wireless photonic and contracted implantable devices for light delivery into deep regions, such as the brain and liver, rendered PDT treatment even more effective [62].

### 2.5. Effect of Light on NF-кB Activation and ROS Regulation.

Activation of NF-кB depends on various parameters, like the cell compartment and dimer confinement. Cytoplasmic NF-кB dimers are maintained in a stationary and inactive state through their association with IкB (inhibitor of κB) proteins. When unconfined, NF-кB dimers translocate to nuclear regions and bind to targeted DNA sequences, promoting gene expression [63]. Other determinant factors include the synthesis of IкB proteins, activities of IKK (IκB kinase) complexes, and displacement of NF-кB family members, both transcriptional co-activators and DNA [64]. The N-terminal Rel homology domain (RHD) has affinities to кB sites and characterizes members of the NF-кB family, consisting of RelB, RelA/p65, c-Rel, p50 (NF-кB1), and p52 (NF-кB2) in mammals [65]. The binding affinities of their кB sites are of critical merit to exert positive and negative effects on transcription processes, dimerization formation, and maintenance [60]. Only p65, RelB, and c-Rel mediate transcription through their C-terminal transactivation domains (TADs). Though they have no TADs, through interactions with non-Rel proteins with transactivation ability and hetero-dimerization with TAD-containing NF-кB subunits, NF-кB1 and NF-кB2 upregulate transcription on the one hand and downregulate transcription on the other hand via competitive inhibition with TAD-containing dimers [66]. 

**Figure 3:**
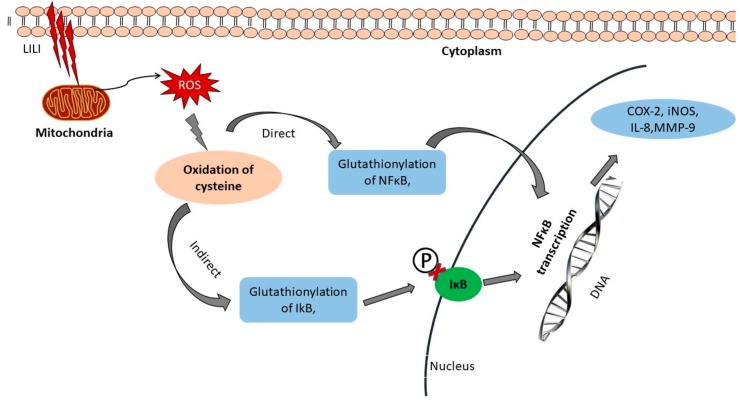
Mechanism of photobiomodulation (PBM). When low-powered light (LILI) enters the cell, it is immediately absorbed by cytochrome c oxidase, which is present in mitochondria. This results in an increased respiratory chain reaction and the overall redox reaction is altered. The increase in reactive oxygen species (ROS) stimulates the oxidation of cysteine molecules. The glutathionylation of IkB and S-glutathionylation of p50 activates NF-kB and transcription of specific genes. This further stimulates the activation of the IkB kinase complex, leading to phosphorylation, ubiquitination, and degradation of IkB proteins. Released NF-kB dimers then translocate into the nucleus, bind to specific DNA sequences, and promote the transcription of target genes such as interleukin (IL)-2, IL-6, IL-8, inducible nitric oxide synthase (iNOS), COX-2, and matrix metallopeptidases (MMP)-9.

Activation of NF-κB can be observed by direct and indirect glutathionylation of NF-κB and I-κB, respectively. The p50 of cysteine-62 is sensitive to oxidation, which inhibits the translocation of NF-κB into the nucleus and prevents DNA binding [67]. However, as S-glutathionylation events occur, p50 is selectively reduced and DNA binding is restored [68,69]. ROS modification and glutathionylation of IκBα at cysteine 189 prevents phosphorylation events and degradation, thereby leading to NF-кB activation and targeted gene transcription (Figure 3) [70]. The IкB-mediated regulation characterizes the NF-кB pathway and IкB proteins (IкBα, IкBβ, IкBε, IкBz, B-cell lymphoma 3 (BCL-3), and IкBns), together with the precursor proteins NF-кB1 and NF-кB2, are well defined by the presence of multiple ankyrin repeat domains. NF-кB activation, via phosphorylation of IкBs on conserved serine residues, facilitates recognition by bTrCP proteins and K48-linked polyubiquitination by the Skp1–Culin–Roc1/Rbx1/Hrt-1–F-box family of E3 ligases acting alongside the E2 enzyme UbcH5 in a precise manner [63]. The NF-кB signaling pathways are classified into canonical and non-canonical routes. The canonical route is a classical representation of the generalized regulation of the NF-кB pathway. Upon ligand recognition, cytokine receptors, such as the antigen receptors, interleukin (IL)-1 receptor (IL-1R), tumor necrosis factor receptor (TNFR), and pattern recognition receptors (PRRs), stimulate and activate signaling cascades that culminate in the activation of IKKβ (IKK2). IкB proteins are phosphorylated by activated IKKβ, which exists in a complex with the closely related kinase IKKα (IKK1) and NF-кB essential modulator (NEMO, IKKγ). Many membrane-bound ligands, such as TNFR, TLR, and IL-1R, are involved in activation of the NF-кB pathway and act as upstream regulators. When activated, the IKK complex becomes the principal upstream part of all NF-кB pathways, thereby regulating the transcription of proinflammatory genes [71]. 

**Figure 4:**
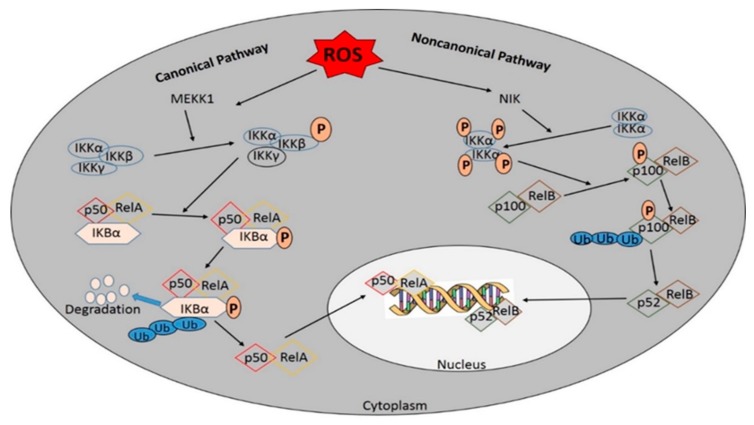
Activation of NF-кB by the canonical and/or the noncanonical pathway. The canonical NF- кB-activating pathway depends on the phosphorylation of IкB-kinase (IKK) β. The phosphorylation and ubiquitination of IкBα translocate NF-кB into the nucleus. where it helps in the transcription of target genes. Many membrane-bound ligands involved in activating the NF-кB pathway act as effective upstream regulators and the IKK complex is the common upstream component of all NF-кB pathways. In contrast, the non-canonical pathway is NF-kB essential modulator (NEMO)-independent and depends on IKKα activation to induce the NF-kB/RelB activation complex, leading to the phosphorylation of p100 and the generation of p52/RelB complexes.

The archetypical IкBα protein promptly degrades during the canonical activation route, causing the release of multiple NF-кB dimers. IкBα principally targets the p65:p50 heterodimer to form a IкBα: p65:p50 complex, which has translocating capabilities and masks the p65 nuclear localization signal. DNA binding is prevented by the IкBa nuclear export signal, which results in steady-state cytoplasmic localization of NF-кB dimers [72]. The degradation of IкBα leads to localization of NF-кB in the nucleus and removal of IkB from the NF-кB complex. Then, NF-кB dimers bind to specific DNA кB sites in the promoters and enhancers of target genes, leading to the formation of homodimers and heterodimers, and heterotypic interactions with other transcription factors, finally leading to regulation of transcriptional activity via post-translational modifications of targeted NF-кB subunits [66]. The termination of transcription depends on the synthesis of typical IкB proteins and the elimination of active NF-кB dimers from DNA. The difference between canonical and non-canonical routes remains whether they are NEMO-dependent or NEMO-independent. The non-canonical pathway is NEMO-independent and is determined by IKKα activation to induce the NF-kB/RelB activation complex, thereby leading to the generation of a p52/RelB complex and phosphorylation of p100 (Figure 4). IKKα proteins can activate the canonical route and expand to the non-canonical route through the induction of p100 expression [73].

### 2.6. Reciprocal Influence of ROS and NF-кB Activation

Hydrogen peroxide inhibits IKK activation by directly affecting IKKβ through its cysteine inhibitory ability in catalytic domains of tyrosine phosphatases. The interactions of ROS with cysteine residues are essential in order to affect the NF-кB pathways [74,75]. They facilitate the phosphorylation of serine residues in the activation loops of IKK in cells [75], and IKK triggers the ubiquitination and degradation of IkBα, which is usually phosphorylated on serine 32 and 36 [76]. The kinase upstream of IKK or mitogen-activated protein kinase 1 (MEKK1) is inactive when glutathionylated at C1238. ROS also target IKK and affect the NF-кB pathway, S-glutathionylation and IKKB functions via phosphorylation and degradation of IкBα on tyrosine residues and activation of the NF-кB pathway, as this may inhibit IкBα phosphorylation. They have the potential to influence the ubiquitination and degradation of IkB and activate NF-кB by inactivating Ubcl2 [77]. In the non-canonical route, NF-кB-inducing kinases (NIK), i.e., the upstream kinases, are activated by ROS through inhibition of phosphatase and oxidation of cysteine residues [78].

Moreover, ROS appear as vital bridging factors that mediate the crosstalk between JNK and NF-кB signaling pathways and interconnect ROS-mediated NF-кB and cell death, with the ability to inhibit JNK activation [79]. Furthermore, NF-кB downregulates JNK activation by suppressing TNFα-induced ROS accumulation. Due to their exerted oxidative stress, ROS directly participate in upregulation or downregulation of NF-кB through their interactions with cytoplasmic components. ROS are noxious at certain cellular levels and their inhibition results in the blockage of TNFα-induced programmed cell death facilitated by the NF-кB signaling pathway [80]. However, nuclear ROS might solely lead to a reduction of the binding ability of NF-кB to DNA [81]. The respective ROS accumulation and resulting oxidative stress generated in a specific subcellular localization becomes significant, as oxidative damage could be avoided and cell survival could be maintained [82]. Cell survival is the most probable outcome of the increased expression of NF-кB target genes, and only in a few exceptional cases does a cell death response prevail. Therefore, it is expected that, on one hand, ROS would modulate NF-кB responses, and on the other hand, NF-кB target genes would promote survival by ROS attenuation [83]. 

### 2.7. NF-кB in Cancer Progression 

NF-кB is involved in various stages of oncogenesis and controls the expression of certain tumor-related genes. During initiation in pre-malignant cells, stimulated NF-кB promotes the expression of cytokines and chemokines, leading to the recruitment and activation of immune cells, which produce more chemokines and growth factors [71]. They act on both malignant and inflammatory cells in an autocrine or paracrine way, creating a complex inflammatory and pro-tumorigenic microenvironment. NF-кB mediated inflammation contributes to nuclear damage, oncogenic mutation, and tumor initiation and progression in pre-malignant cells [84]. Epidemiological studies revealed that over 20% of all cancer types are associated with chronic inflammation, which triggers carcinogenic events, leading to the malignant transformation of normal cells [85]. Abnormal NF-кB activity may promote the induction of tumors and apoptosis-resistant genes; this is one of the mechanisms by which resistance to radiotherapy and chemotherapy is acquired. Through cytidine deaminase enzymatic activity, activation-induced cytidine deaminases (AID) cause genetic alterations in DNA sequences, and their intrinsic mutagenic-enzyme forms can be induced in response to infectious agents and pro-inflammatory cytokines that facilitate NF-кB activation in epithelial cells [86]. While the major role of NF-кB in the regulation of AID expression is evident, NF-кB-mediated AID expression is accomplished via inflammatory mechanisms for the malignant transformation of epithelial cells during carcinogenesis. In normal cells, NF-кB is transiently activated, while in cancer cells, it exhibits sustained activation [87]. 

**Figure 5:**
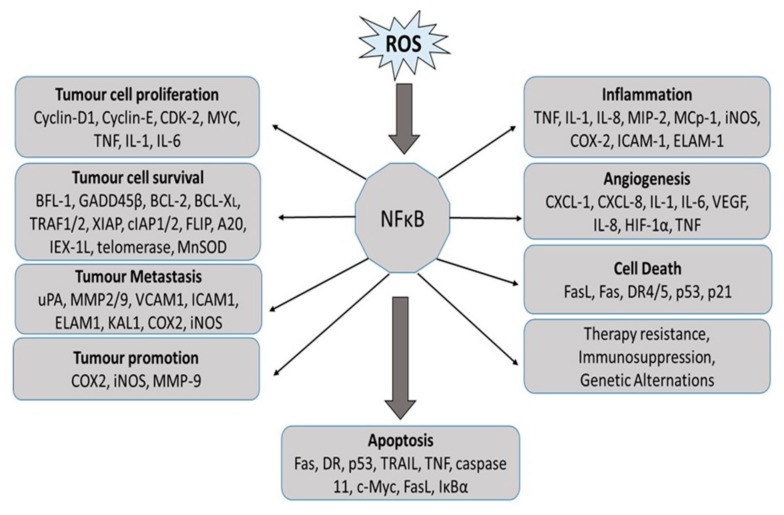
NF-κB activation in the progression of cancer by regulating genes involved in tumor cell proliferation, cell growth, survival, angiogenesis, tumor promotion, and metastasis. BCL2: B-cell lymphoma protein 2; BCL-XL, also known as BCL2-like 1; BFL1, also known as BCL2A1; CDK2: cyclin-dependent kinase 2; COX2: cyclooxygenase 2; CXCL: chemokine (C-X-C motif) ligand; DR: death receptor; ELAM1: endothelial adhesion molecule 1; FLIP, also known as CASP8; GADD45beta: growth arrest and DNA-damage-inducible protein beta; HIF1alpha: hypoxia-inducible factor 1 alpha; ICAM1: intracellular adhesion molecule 1; IEX-1L: radiation-inducible immediate early gene (also known as IER3); IL: interleukin; iNOS: inducible nitric oxide synthase; KAL1: Kallmann syndrome 1 sequence; MCP1: monocyte chemoattractant protein 1 (also known as CCL2); MIP2: macrophage inflammatory protein 2; MMP: matrix metalloproteinase; MnSOD: manganese superoxide dismutase (also known as SOD2); TNF: tumor necrosis factor; TRAF: TNF receptor-associated factor; uPA: urokinase plasminogen activator; VCAM1: vascular cell adhesion molecule 1; VEGF: vascular endothelial growth factor; XIAP: X-linked inhibitor of apoptosis protein.

NF-кB is involved in tumorigenesis by upregulating the anti-apoptotic pathway and tumor cell survival. The canonical pathway of NF-кB is known to activate the transcription of a group of anti-apoptotic proteins, which is further subdivided into two categories, namely, inhibitors of apoptotic proteins and the Bcl-2 family members [88]. In addition to transcription, inhibition of NF-кB activity promotes JNK activity and apoptosis, suggesting that NF-кB inhibits apoptosis via inhibition of JNK activity [89]. Pro-survival functions of NF-кB are related to the expression of phosphoinositide 3-kinase (PI3K/Akt cascade), one of the key elements in promoting cell proliferation and growth [90] (Figure 5).

### 2.8. NF-кB in the Pathogenesis of Diabetes

**Figure 6:**
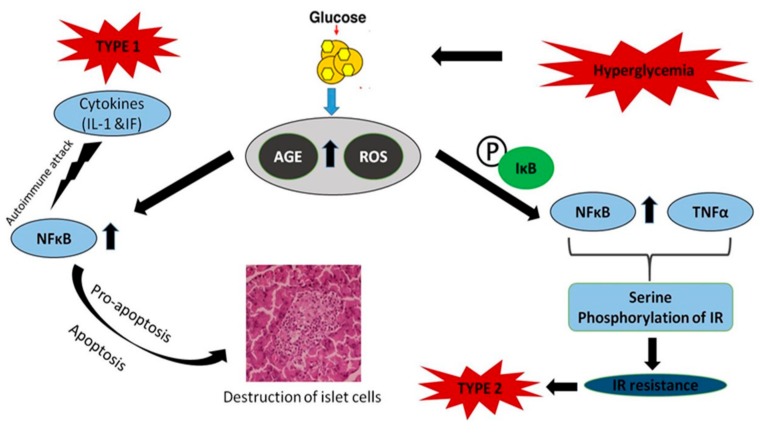
Role and activation of NF-кB in diabetes mellitus. NF-кB regulates the expression of cytokine-induced genes that affect pro- or anti-apoptotic cascades. NF-кB is one of the major causes in the development of insulin resistance. Tumor necrosis factor (TNF) predominantly induces insulin resistance by the serine phosphorylation of insulin receptor substrate-1 (IRS1), and NF-кB is a major cause of insulin resistance.

Insulin-dependent (type 1) and insulin-independent (type 2) diabetes are the two major variations of diabetes seen among humans. In type 1 diabetes, beta pancreatic cells are prone to autoimmune attack by cytokines, such as interferon and IL-1, and NF-кB is mostly inactive in resting cells. However, IL-1 can activate the translocation of NF-кB to the nucleus [91]. NF-кB regulates the expression of cytokine-induced genes, which play very significant roles in pro- or anti-apoptotic cascades (Figure 6). Cytokine-induced NF-кB activation can be reverted by a non-degradable mutant form of inhibitory кB (IкB) (S32A, S36A) in recombinant adenovirus (AdIB (SA) 2) in human pancreatic islet cells or by transfection with a stable inhibitor of NF-кB in purified rat cells [92,93]. Moreover, intravenous administration of a “dummy” NF-кB and mice deficient in NF-кB (p50) showed resistance to alloxan- and streptozotocin-induced islet cell death [94]. This could be considered a strategy to protect cytokine-induced in vitro and in vivo apoptosis, whereby inhibition of NF-кB is achieved. This highlighted the pro-apoptotic effect of NF-кB activation in β-cells of the pancreas. Blocking NF-кB for a prolonged period displayed impaired development of endocrine cells and expression of important genes involved in the insulin-secretion pathway [95]. Interestingly, resistance to NF-кB activation could decrease the time course of the development of diabetes. Hence, it is more important to consider the mechanisms that regulate NF-кB in cells and genes in diabetic conditions before building up inhibition strategies.

Type 2 diabetes is mainly characterized by insulin resistance. NF-кB is one of the chief components in the insulin resistance observed in type 2 diabetes. The anti-inflammatory agent aspirin prevents the degradation of NF-кB inhibitor (IB), whose expression in the liver attenuates the expression of NF-кB dependent genes, but also reduced the likelihood of type 2 diabetes development. Similarly, inflammation-induced hyperglycemia can be inhibited using specific inhibitors of interleukin-1 signaling [96]. Hence, it is obvious that the target genes of NF-кB, such as IL-1, TNF, and IL-6, and NF-кB itself, play vital roles in insulin resistance. NF-кB also antagonizes peroxisome proliferator-activated receptor (PPAR), which maintains glucose homeostasis in bone marrow-derived mesenchymal cells [97]. TNF predominantly induces insulin resistance by serine phosphorylation of insulin receptor substrate-1, which is also a potent activator of NF-кB (Figure 6). Evidence suggests that TNF is involved in insulin resistance in humans and animal adipose tissue and blocks reversion of the diabetic condition by masking the activity of NF-кB [98]. Hence, NF-кB is one of the major causes in the development of insulin resistance. Given the role of NF-кB in insulin regulation, it is important to understand its beneficial effects in insulin sensitivity and GLUT2 monitoring, a protein that controls glucose-stimulated insulin secretion by cells [95]. In certain cases, inhibition of the transcription factor NF-кB may inflict deleterious effects on GLUT 2, leading to diabetes progression. Although there are a lot of studies highlighting the role of NF-кB in type 1 and 2 diabetes, its specific trail in the pathogenesis of diabetes requires further investigation.

### 2.9. NF-кB in Wound Healing

NF-кB is essential for inflammatory and phagocytic cell migration as it strictly regulates genes involved in cell proliferation (granulocyte colony-stimulating factor and macrophage colony-stimulating factor), transformation, and survival. In wound healing, the classical NF-кB pathway is activated as an innate immune reaction and, as a result, activation of NF-кB is more important in the protection of cells from infections or microbes [63]. The effects of NF-кB on cell survival mainly depends on the type and number of stimulatory molecules [99]. In normal wound healing responses, NF-кB functions as a positive signaling molecule to enhance the proliferation phase, and both increase and regulate the migration of keratinocytes toward re-epithelization sites [100,101]. Under normal physiological conditions, NF-кB protein levels are standard, hence there is no defined physiological level to clear dead cells and microbes during responses to infections or wounds. Any defect in NF-кB activation inhibits the innate immune reaction, whereas the elevated production of NF-кB increases inflammatory cytokine levels and leads to tumor formation [102]. Every wound is different and requires integrated defence and repair mechanisms. In clinical settings, wound evaluation cannot solely depend upon Nrf2 and NF-кB but can indicate an inadequate defence against oxidative stress or innate immune reactions at large in non-healing wounds. For wounds with prolonged inflammation, compounds that decrease NF-кB levels or activate the Nrf2 pathway can be useful, and Nrf2 and NF-кB as markers are mainly utilized to assess healing processes. In immunosuppressed patients, NF-кB induction can help to initiate the correct immune reaction, but the multifaceted network of active molecules in the whole body can cause a different cell response than that observed in vivo [103]. 

## 3. Conclusions

Unusually high levels of ROS can allegedly be used for cancer diagnosis, varying according to tumor type, and are potent signaling molecules in cancer scenarios, thereby leading to nuclear damage, genetic instability, and tumorigenesis. The regulation of redox transcription factors is dependent on the generation of ROS levels. NF-кB was the first redox-regulated transcription factor to be discovered. It is a protein complex with multiple functions in immune, inflammation, cell growth, and survival responses. ROS have NF-кB stimulatory effects in the cytoplasm and inhibitory effects in the nucleus. Therefore, ROS have the ability to both activate and suppress NF-кB signaling pathways. PBM promotes photon availability and absorption by chromophores in the catalytic center of mitochondrial cytochrome c oxidase the and subsequent reduction of molecular oxygen. This disturbs the mitochondrial membrane potential and changes the fission–fusion homeostasis in the mitochondrial network, leading to increased levels of intermediates such as ATP, cAMP, and ROS. PBM-mediated ROS interact with certain family proteins that enclose highly reactive cysteine (Cys) residues and after the oxidation of Cys residues. The activity of NF-кB is regulated by direct activation or after disassembling from IkB, an inhibitor, then the released NF-кB translocate to the nucleus and promotes gene transcription. The regulation of crosstalk between ROS and NF-кB appears to be a key factor for better management of many conditions. PBM facilitates the reduction of oxidative stress and activation of mechanisms that upregulate or downregulate NF-кB, which are critical for the promotion of anti-apoptotic responses and tumor progression, insulin dependence or resistance in diabetes, cell proliferation and migration in wounded sites, or cell survival by ROS attenuation.
